# Community as the teacher on issues of social responsibility, substance use, and queer health in dental education

**DOI:** 10.1371/journal.pone.0237327

**Published:** 2020-08-14

**Authors:** Mario Brondani, Maxine Harjani, Michael Siarkowski, Abiola Adeniyi, Krista Butler, Sekani Dakelth, Russell Maynard, Kinnon Ross, Cormac O’Dwyer, Leeann Donnelly

**Affiliations:** 1 Department of Oral Health Science, Faculty of Dentistry, University of British Columbia, Vancouver, British Columbia, Canada; 2 Megaphone Speakers Bureau, Vancouver, British Columbia, Canada; 3 Portland Hotel Society - Community Services, Vancouver, British Columbia, Canada; 4 Providence Health Care, Vancouver, British Columbia, Canada; 5 Stigma and Resilience Among Vulnerable Youth Centre, University of British Columbia, Vancouver, British Columbia, Canada; 6 Department of Oral Biological & Medical Sciences, Faculty of Dentistry, University of British Columbia, Vancouver, British Columbia, Canada; University of Queensland, AUSTRALIA

## Abstract

**Introduction:**

In order to foster dental and dental hygiene practices that are inclusive, sensitive to diversity, equitable, and without prejudice, a call to broadly teach cultural diversity within dental and dental hygiene education has been made. The research question of this study was “to what extent can an interactive and open dialogue about substance use, queer health, and social responsibility foster transformative learning?”

**Methods:**

A collaborative and interdisciplinary project engaged *the community as a teacher* over the Summer and Fall of 2019 to address issues of substance use, queer health, and social responsibility and was delivered to 55 first-year undergraduate dental and 23 third-year dental hygiene students over three educational sessions. Dental and dental hygiene students were asked to reflect, in writing, on each session using between 200 and 400 words. Textual information from students’ self-reflections and from the community’s feedback were analyzed thematically for content (e.g., codes and themes).

**Results:**

In total, 128 written reflections–for an average of 42 reflections per session–were gathered and analyzed interactively by the authors. Three major themes emerged: feeling privileged, breaking stereotypes, and coalescing learning. Feedback from the participating community members highlighted changes to be implemented in these sessions in the future, including more opportunities for small group activities in class.

**Conclusions:**

The three major themes that emerged from the thematic analysis of the self-reflections and community member feedback (feeling privileged, breaking stereotypes, and coalescing leaning) further highlighted the impact of community-driven curricula on students’ learning in regard to substance use, queer health, and social responsibility. Further work is critical to understand the impact of such a pedagogy on students’ practices once they leave their undergraduate programs.

## Introduction

Despite the associations between oral health and general health, dental care is not part of a universal, nation-wide health care system in many countries, including Canada, and remains at a prohibitive cost to many [1]. Those who cannot afford the costs also bear most of the treatment needs [2]. In particular, individuals suffering from substance use, along with some members of the *queer community* (an umbrella term collectively referring to many variants of gender identity and sexual orientation) [3], might be vulnerable and marginalized, and face further barriers in assessing much-needed dental care. Although affordability is the main reason vulnerable and marginalized individuals do not access dental care, they are often also reluctant to seek care because they fear judgement, rejection, and stigmatization by dental care providers–outcomes previously felt among those with a history of substance use and mental illnesses [4], living with HIV [5], experiencing homelessness [6], and with a history of incarceration [7]. Despite professional efforts to engage in charitable dental care services for those in need [1, 8], the concept of a socially responsible dental professional able to counteract the prevailing oral health disparities and to broaden access to oral health care to those most in need has been questioned [9]. Concurrently, queries have arisen as to whether or not it is possible to educate future oral health care professionals on how to be socially responsible [10].

Nonetheless, within the idea of being socially conscientious, attempts have been made to reduce stigma and discrimination within the health care system. However, these negative experiences remain very visceral for many individuals seeking oral health care services [4–6]. In order to foster dental and dental hygiene practices that are inclusive, sensitive to diversity, equitable, and without prejudice, a call “to teach cultural diversity” [11] broadly within dental and dental hygiene education has been made. In the era of globalization, teachings related to substance use [12], queer health [13], and social responsibility [10] need to be revisited within alternative pedagogies beyond lectures, and within a flipped classroom approach by introducing students to learning material before class and deepening their understanding of the content within classroom time [14, 15]. Moreover, pedagogies in which the community is engaged as a teacher should be favored, adding depth and context to a solely community-based dental education where students are enabled to get a positive learning experience while providing needed dental services to the underserved [16]. Community-engaged learning not only addresses community-identified concerns and their context, but also utilizes the expertise of the community in proposing, contextualizing, and delivering the didactic content and guiding any community experiences so that the teaching is culturally appropriate and meaningful from their own perspectives [17].

Whether in the community or in the classroom, written self-reflections are highly encouraged so students can ponder what, and how, they learn in a safe and non-judgmental environment [18]. When students are encouraged to reflect upon an experience, a lesson learned, a challenge faced, or on the impact of an activity [19], the exercise enhances knowledge and facilitates personal and professional development [20]. By combining the “community as a teacher” with written self-reflections we were able to develop the research question of this study: To what extent can a community-driven, interactive, and open dialogue session about substance use, queer health, and social responsibility foster transformative learning? This research question led to the following objectives: to identify, develop, deliver, and evaluate an educational curriculum to address key issues of substance use, queer health, and social responsibility within the first-year undergraduate dental and third-year dental hygiene students.

## Methods

Approval for this research was obtained from the Behavioural Research Ethics Board (#H19-01005) at the University of British Columbia (UBC). The community advisory group (K Butler, S Dakelth, R Maynard, K Ross, and C O’Dwyer) was formed from existing contacts the Faculty of Dentistry had cultivated in the community over the years, with representatives from a number of organizations. This community advisory group and the research team convened face-to-face on two occasions and a number of times via electronic communication to discuss the curriculum’s content and to inform the process of delivering and evaluating the content (Table 1). These meetings and information exchanges occurred over the summer and fall of 2019, and during the winter of 2020, with meetings alternating between the university campus and host community sites. Student input was further obtained through class representatives from the dental (M Siarkowski) and dental hygiene (M Harjani) programs, as well as from the graduate level (A Adeniyi), to determine common interest areas. These three students were selected as they have been working with the research team on other projects and showed interest in taking part in the study described herein.

**Table 1:** Content, delivery methods and scheduling, and suggested evaluation strategies to address substance use, queer health, and social responsibility within undergraduate dental education.

	Substance use	Queer health	Social responsibility
**Pre-session**	➢ Online anonymous question^ to students: *“What would you like to learn from this session*?*”*		
	➢ Pre-reading:	➢ 10-min video:	➢ Pre-reading:
	*Integrating issues of substance abuse and addiction within undergraduate dental education* [13]	*An introduction to the Queer community* [3]	*Teaching social responsibility through community service-learning in predoctoral dental education* [10]
**2.5 hrs session (2:30-5PM)**	➢ Community driven Workshop^>^ (100 min)	➢ Community driven presentation* (50 min)	➢ Community driven presentation (90 min)
	➢ Individual exercise (20 min)	➢ Q&A with community members (60 min)	➢ Case study discussion in pairs (30 min)
	➢ Personal testimonial of community members (30 min)	➢ Case study discussion in pairs (40 min)	➢ Personal testimony of community members (30 min)
**Post-session**	➢ Self-reflection:^#^		
	“*How has this session on substance use/queer health/social responsibility informed you on the practice of an inclusive and socially responsible dental and dental hygiene practice*?”		
**Evaluation strategy**	➢ Number of students answering the learning outcome question		
	➢ Content and depth of suggested learning outcomes		
	➢ Number of reflections submitted		
	➢ Content and depth of the reflections submitted		
**Debrief**	➢ Debrief sessions with the participating community members and the academic researchers following the respective sessions		

^Question was posed online via the University of British Columbia Qualtrics anonymous survey tool.

^>^Workshop run by the Speakers Bureau, from Megaphone Magazine, dedicated to de-stigmatization training.

*These presentations were run by members of the respective community they represent.

^#^Students were asked to reflect freely and respond anonymously also using the University of British Columbia Qualtrics survey tool; the word limit was set at 400 words.

The community advisory group and the research team planned three 2.5-hour community-driven sessions to separately cover topics of substance use, queer health, and social responsibility (Table 1). Three sessions were delivered to the entire class of 55 first-year dental students and 23 third-year dental hygiene students in the January 2019/20 academic year at UBC’s Faculty of Dentistry. These sessions were mandatory for the undergraduate students from both dentistry and dental hygiene programs. Table 1 presents the topics covered in each of the sessions according to the existing literature on substance use [4, 12], queer health [10], and social responsibility [13]; they also had a pre- and post-session component, interactive presentations from the respective communities, and in-class individual, paired, and group activities. Each session was delivered only once, during the first three Wednesdays in January. This study reports on the thematic analysis of the data obtained from guided self-reflections the students provided while answering the following question posed in each of the sessions described in Table 1: “*How has this session (on substance use/queer health/social responsibility) informed you on an inclusive and socially responsible dental and dental hygiene practice*?” Reflections were posted via the University of British Columbia Qualtrics tool, ranged from 200 to 400 words each, and were de-identified for the thematic analysis described ahead. The inclusion criterion was that the reflections were submitted by the students who took part in the three mandatory sessions. Unlike participation in the three sessions, reflections were encouraged but not compulsory. We also report on the feedback gathered from the community in the form of post-session debriefs, where free-written notes were taken by the first author.

Textual information (students’ self-reflection and the community feedback’s notes) were analyzed thematically for content by two authors (LD and MB) via a coding process. Thematic analysis was used so the authors could closely examine the data to identify common themes in the form of ideas and patterns of meaning via coding. Coding refers to a single word in the form of a verb, noun, or adjective that is attached to a sentence or sentences in the text of each reflection judged to be convening specific content; these codes were created by the two authors (LD and MB) individually, as they have done in other studies [4, 13, 18, 19]. This interactive coding process helped the authors identify repeating ideas within, and between, the written self-reflections and feedback, which were then grouped into emerging themes; such a process also enabled increased rigor of the study via an audit trail, with the two authors coding the same textual data and using similar denominations (i.e., codes) as they met to discuss the coding process until consensus was reached. One of the authors (MB) then carried out the remainder of the thematic analysis, which was revisited and discussed with the other authors for input. Excerpts from quotes are used to illustrate the themes that emerged on the issues of substance use, queer health, and social responsibility from these community-driven dental education sessions. Because the reflections were de-identified, the information about gender, age of the student, or if they were from the dental or dental hygiene program remains unknown.

## Results

In total, 128 written reflections were gathered from both the dental and dental hygiene students, for an average of 42 reflections per session. Some students may have submitted reflections for all three sessions, while some might not have submitted reflections for any session; it is unlikely that the same student would have submitted more than one reflection for the same session given the way Qualtrics is set up, as responses are attached to a particular computer IP address. Free-written notes from four meetings with the community advisory group were also gathered. Three major themes emerged from the analysis: feeling privileged, breaking stereotypes, and coalescing learning. We present and expand on each of these themes below.

### Feeling privileged

Some students felt fortunate to have experienced the delivery of this content by experts, i.e. the community itself, either during the session on substance use: *“it was a privilege to have this session delivered by the Megaphone group who put their faces out there*, *in front of us with pride and yet*, *being humble and understanding”*; or during the session on social responsibility: *“this session gave me the opportunity to hear from that side of the community and what they want from a socially responsible dentist—the other side is the dental professional recognizing [their] privilege and giving back what they think society needs”*.

Other students commented on the fact that they were privileged for “*having a stable family*, *for studying at a university pursuing a professional degree*, *for having had opportunities in life”* when reflecting on the personal struggles some of the community members shared. The concept of privilege was also echoed when some students reflected on the responsibility they have for the care they are learning to provide. For example, one student’s reflection for the pre-reading on queer health session stated that “*as health care professionals*, *we are granted by the regulatory authority the right to be part of a restricted group*, *of a group of people that will be responsible for caring for others regardless of how they look*, *what they do*, *or who they are”*. Privilege was also understood as empowering yet overwhelming, as mentioned by one student when referring to their duties as a health care provider: *“it is really empowering*, *and a tremendous and serious role we have if we think about the privilege we will hold to care for somebody who will trust their care to us”*.

### Breaking stereotypes

When community members experiencing issues at hand share their stories, it can be transformative to those listening. In these particular sessions, the shared stories also helped to break stereotypes. One student wrote: “*this session changed the way I look at somebody panhandling from now on…I always held negative stereotypes about them*, *and today it was an opportunity to start changing that”*, while another student commented that “*we are all people*, *and things can happen to any of us*. *Who are we to judge somebody else*?*”*

On the issue of substance use, one student questioned the idea of abstinence while discussing carrying out daily life activities: “*we hear all the time that drug use is bad* … *the fentanyl crises is here to confirm that*, *but it was really interesting to hear that Kris* (community presenter currently using illicit drugs) *still uses drugs to be functional*, *to perhaps help him to forget the trauma he suffered before*. *If he abstained*, *he would not be able to cope”*. The suggested pre-readings and videos (Table 1) were also instrumental in helping students break stereotypes. In particular, the video presenting and defining different acronyms used to refer to the queer community made some students realize that *“people are not gay or straight … they can be many things and take many forms outside this idea of black and white*. *Even more mind-blowing is to know that sexuality is a spectrum*, *[and] changes overtime to some”*; this video also helped one student *“appreciate that there is a wide range of people from different life paths*, *who do not conform with the ‘norm’ and who stay true to themselves”*.

### Coalescing leaning

As educators, we strive to make learning relevant to the student and better connected to the rest of the curriculum. The participation of the community helped to coalesce learning, not only from the suggested pre-readings, but also from other modules and sessions of the curriculum outside these three sessions. This was particularly relevant to Indigenous content, that, according to one student “*came together…the stories presented added realism to addiction*, *and the First Nation presenter brought an Indigenous lens to the discussion and tied up nicely with the Interprofessional Education activities we had last term”*. With regard to the session on social responsibility, another student brought up the social determinants of health in their reflection: *“last term we heard about the social determinants of health and it was a bit ‘dry’… but after today*, *it was clear how things in life can impact your trajectory and how trauma*, *abuse*, *lack of opportunity*, *and living arrangements can drive your health to a less desirable outcome”*. For another student, the learning came together even if one aspect of their dental training was still missing: *“we have not seen a patient for clinical care yet*, *but the session today reinforced the idea that we have to treat every patient equally regardless of their gender identify or status*, *or their choices in life”*. For others, these sessions gave them the opportunity to reinforce what they already encountered in their training: *“we go to the community every week*, *and we see the cases portrayed within the stories we heard today* … *but it was great to hear those stories shared out loud with pride”*.

For at least seven students, sessions like these might not have been as inspiring or have had the same impact as that felt by the majority of the other students; they may also have interpreted the stories differently. One student, for example, questioned the potential for normalizing substance use by hearing from a community member that still uses substances to function: *“I don’t think we should normalize drug use … is there such a thing as a functioning alcoholic performing surgeries*?*”*, while another student raised the issue of other addictive behaviors not being addressed in class: *“there are other addictions that destroy lives and careers … gambling for example*, *and yet*, *TV ads for online casino[s] are all over and we do not talk about it”*. For one student reflecting on the social responsibility session, personal student debt came into play when considering giving back to society: *“I think that we are preaching to the choir when talking about reaching out to those in need*. *As dentists these days*, *you are kind of expected to give back in one way or another*, *but how can you do that with a debt of more than $250 thousand dollars just from your education*, *let alone the cost of your own practice*?*”*

In terms of feedback from the participating communities, ideas surfaced mostly regarding changes to be made if these sessions are to be offered again in the next academic year. For example, we were told to “*include a discussion about harm reduction and how it relates to the sessions*, *particularly the substance use one”*, to *“invite a variety of other community members who could give students a different view on other issues faced by the community”*, and to *“encourage small group discussion*, *together with the exercise in pairs where students are presented with short case scenarios* (to be developed) *so that the content can better be assimilated”*. The community members that delivered the sessions also praised the flipped classroom approach that, according to them, *“prompted discussions straight to the point as we did not have to spend too much time in explaining some of the terminology and concepts”* and enabled “*students to have very good questions based on the material they were given before the class*”.

## Discussion

Our research question, “to what extent can a community-driven, interactive, and open dialogue about substance use, queer health, and social responsibility foster transformative learning?”, highlighted that this educational method for use in undergraduate curricula can indeed have a positive impact on students’ learning. The thematic analysis identified that students questioned the idea of privilege, broke down their own personal stereotypes, and brought together previous learning in a meaningful way that went beyond what can be learned through academic literature and textbooks alone. By not requiring a more detailed theoretical approach compared to other qualitative inquiries, the thematic analysis provided a highly flexible style that offered a rich and detailed, yet complex account of textual data [21] pertaining to community-driven education. This type of education also recognizes the expertise that community members have in regard to the content, how to best contextualize and deliver it, and what is important in meaningfully evaluating such activities. The use of reflective learning has been an important component of undergraduate dental education at UBC’s Faculty of Dentistry for decades [17]. Self-reflection has long been discussed as a positive and engaging activity; our own studies have already illustrated the impact of such learning tools, particularly within a community-service learning-based curriculum [17, 18]. The difference lays in the fact that within these three sessions, the community was not the recipient of the care (as found in a traditional service-learning approach), but was instead recognized as content experts and as the instructor in the classroom; the learning happened organically since course content was identified, contextualized, developed, and delivered collaboratively.

Although we have been delivering sessions on queer health [10], substance use [9], and social responsibility [11] since 2008, both the dental and dental hygiene programs have since changed to comply with renewed accreditation requirements over the past decade. For example, since 2015/16, under the umbrella of UBC Health, dental and dental hygiene students receive part of their education by joining other health professional programs in an integrated educational module covering six themes [22]—one being Indigenous Health, which was mentioned in some of the reflections. This integrated educational curriculum is relatively new and has had an impact on the amount of content both programs must address. It has also impacted the knowledge disseminated from different areas of the curriculum, as reflected by the students. Moreover, the #MeToo and Time’s Up movements, the #BlackLivesMatter global network to fight abuse and racism, the legalization of marijuana, the opioid/fentanyl crisis, and various campaigns to destigmatize substance use have shaped many facets of our society in recent years, particularly in Canada. As such, it was necessary to deliver these session topics via more engaged community-driven education, and the community was involved since the inception of this re-designed educational curriculum. The educational project in which these sessions took place offered ways to engage with equity, diversity, and inclusion in teaching and learning environments for the benefit of students by using a blended learning/flipped classroom approach. As suggested by Vanka and colleagues, the flipped classroom approach offers strategies that adopt and incorporate contemporary teaching methods to keep up with technological advances [23]. As such, the pre-readings and videos (Table 1) were made available to the students before the sessions, so that they could arrive better prepared and with some basic knowledge about the topics. This was indeed appreciated by some of the community members, with feedback stating that “*students have very good questions*”, and by some of the students themselves—with one in particular stating that: “*the information about the session … was intriguing and I was looking forward to hear [about] the experiences from the community members that struggled with substance use”*. While we cannot guarantee that all students read the pre-readings or watched the video before the sessions, we strongly believe that these three sessions have provided a transformative and empowering learning experience for the students.

Feedback obtained from the community attested to the need for constant re-evaluation of educational activities; they suggested a few changes to be considered when these sessions are to be offered in the future, including more in-class time for small group-guided discussions. And although there are many ways to evaluate the actual learning, including formative and summative assessments, the literature appears to agree on the fact that assessment typically drives learning [24]. In other words, students’ *“drive to learn about a subject is directly influenced by the weighting of the subject in the overall assessment”*, as highlighted by Wormald and colleagues [25]. As academics, we have to consider that the lack of direct assessment in terms of grades, and the fact that the reflections were voluntary might have explained the relatively low number of students submitting their written reflective thoughts. We also continue to learn a great deal when working with the community, from recognizing our own privileges as also reflected by the students, to acknowledging and respecting the various ways in which we express feelings and emotions.

Whilst these three sessions had a mostly positive impact, it appears they did not leave the same impression upon all students (as mentioned above); this warrants further studies. As we have emphasized before, any education pedagogy destined to have a positive impact must change students’ attitudes, beliefs, and how they envision their professional responsibility [10–12, 18]. While using the community as a teacher might have had such an impact on most, it may not have been enough to influence all students. Furthermore, we did not follow up with the students after their likely interaction with these various communities during rotations that followed these sessions, so we could not compare their reflections post rotations. Moreover, as we have experienced with the teaching of ethics [26], a community-driven discussion around queer health might not profess to make an otherwise homophobic student queer-friendly [11]; nor will a community-driven conversation around substance use be enough to make an otherwise judgmental student addiction-friendly [10]. However, we still deemed it important to continue offering these sessions, especially with an eye towards social responsibility, such as when those in positions of influence or governance continue to exhibit racist behaviors that go against the commitment to inclusion, justice, and equity [27]. We would also like to engage other dental schools in considering how to use community-driven dental education to teach issues of substance use, queer health, and social responsibility in various dental curricula. Further work is necessary to understand the impact of such a pedagogy on students’ practices once they leave their undergraduate programs.

Limitations of this study include the fact that the three revised sessions were delivered to only one cohort of students from one academic institution; therefore, no comparisons are possible. The voluntary nature of the self-reflections might have limited the number of submissions; although, conversely, it might have encouraged submission by students who were more comfortable in using such means and had something to say. A potential source of bias in the reflections might have come from social desirability, as some students might have felt compelled to write what they think the instructors would like to read. The anonymity of the reflections prevented comparisons based on gender, age, or program–dental or dental hygiene. Lastly, the three major themes presented here are not meant to be exhaustive or to represent all the ideas contained in the 128 self-reflections we received; further analysis is highly recommended.

## Conclusions

The community-planned and driven sessions on substance use, queer health, and social responsibility appeared to foster transformative learning, as they offered an interactive and open dialogue within a flipped classroom approach. The three major themes that emerged from the thematic analysis of 128 self-reflections and the community feedback, including feeling privileged, breaking stereotypes, and coalescing learning, further highlighted the impact of community-driven curricula on students’ learning. Further work is vital to understand the impact of such a pedagogy on students’ practices once they leave their undergraduate programs.
